# Ligninolytic enzymes: Versatile biocatalysts for the elimination of endocrine‐disrupting chemicals in wastewater

**DOI:** 10.1002/mbo3.722

**Published:** 2018-10-17

**Authors:** Ayodeji O. Falade, Leonard V. Mabinya, Anthony I. Okoh, Uchechukwu U. Nwodo

**Affiliations:** ^1^ SA‐MRC Microbial Water Quality Monitoring Centre University of Fort Hare Alice Eastern Cape South Africa; ^2^ Department of Biochemistry and Microbiology Applied and Environmental Microbiology Research Group (AEMREG) University of Fort Hare Alice Eastern Cape South Africa

**Keywords:** endocrine‐disrupting chemicals, laccases, ligninolytic enzymes, manganese peroxidase, versatile peroxidase, wastewater

## Abstract

Direct municipal wastewater effluent discharge from treatment plants has been identified as the major source of endocrine‐disrupting chemicals (EDC) in freshwaters. Consequently, efficient elimination of EDC in wastewater is significant to good water quality. However, conventional wastewater treatment approaches have been deficient in the complete removal of these contaminants. Hence, the exploration of new and more efficient methods for elimination of EDC in wastewater is imperative. Enzymatic treatment approach has been suggested as a suitable option. Nonetheless, ligninolytic enzymes seem to be the most promising group of enzymes for EDC elimination, perhaps, owing to their unique catalytic properties and characteristic high redox potentials for oxidation of a wide spectrum of organic compounds. Therefore, this paper discusses the potential of some ligninolytic enzymes (laccase, manganese peroxidase, and versatile peroxidase) in the elimination of EDC in wastewater and proposes a new scheme of wastewater treatment process for EDC removal.

## 1 INTRODUCTION

EDC are chemical substances that impede the endocrine system leading to negative reproductive, developmental, and neurological impacts in both human and animal (NIEHS, 2010). There exist about eight hundred (800) chemicals suspected to have the capability to interfere with the endocrine system (UNEP/WHO, 2012). Our understanding of endocrine disruption is dependent on clear understanding of the endocrine system (ES). ES is a collection of glands that produce hormones directly into the circulatory system to be transported to target organs, where normal hormonal effects are produced through unified complex signaling pathways involving hormone receptors. The system controls a vast number of biological processes ranging from developmental to functional processes. Any interference in the system will, therefore, result in abnormal hormonal effect, consequently, affecting the development, behaviour, and reproductive system of the organisms (Caliman & Gavrilescu, 2009; You et al., 2015).

EDC either act as agonists or antagonists by their interactions with the hormone receptor. Their mechanisms of action include: mimicking the actions of physiologic hormones, by producing similar physiologic effects; and competitively binding to the hormone receptor, thus, preventing the naturally occurring hormones from binding and, consequently, leading to inactivation and disruption of the hormone synthesis, transport, metabolism, and their corresponding endocrine functions. Major classes of EDC include but not limited to pharmaceuticals and personal care products (PPCPs), phthalates, polychlorinated biphenyls (PCBs), polycyclic aromatic hydrocarbons (PAHs), alkylphenols (APs), alkylphenol ethoxylates (APEs), pesticides including dichlorodiphenyltrichloroethane (DDT), and plastic additives such as bisphenol A (BPA) (Annamalai & Namasivayam, 2015).

Runoff and discharge of treated wastewater effluents into freshwaters are the main sources of EDC contamination, perhaps, due to partial elimination of EDC during wastewater treatment. The receiving waterbodies, which serve as the main sources of portable water, are also used for various domestic and agricultural purposes including irrigation, thus exposing the public to biochemical hazards resulting from poor quality wastewater effluents. The U.S. Environmental Protection Agency has described EDC discharged from wastewater treatment plants (WWTPs) as “contaminants of emerging concern with potentially widespread environmental effects.”

This concern has, consequently, motivated research into EDC including their detection and occurrence in the environment, as well as development of effective method for their elimination in freshwater and wastewater. Recent studies have detected EDC in wastewater (Table 1) and the receiving waterbodies in many countries (Barber, Loyo‐Rosales, Rice, Minarik, & Oskouie, 2015; Komesli, Muz, Ak, Bakirdere, & Gokcay, 2015; Noutsopoulos et al., 2015; Vajda et al., 2015). The micropollutants have also been detected in drinking water and sediments (Liu, Kanjo, & Mizutani, 2009). Some examples of EDC detected in water sediments are nonylphenols (NP), bisphenols, hexestrol (HEX), Diethylstilbestrol (DES), dienestrol, androsterone, *trans*‐dehydrotestosterone (DEHA), 4,5‐α‐dihydrotestosterone (DHT), estrone (E1), 17β‐estradiol (E2), trenbolone, 19‐norethindrone, and 17α‐ethinylestradiol (EE2) (Yuan et al., 2015).

**Table 1:** Detection of EDC in water

Water source	EDC detected	Concentration	Country	Reference
Surface water	Polycyclic aromatic hydrocarbons (PAHs)	Winter: 582.8–2208.3 ng/l Summer: 952.4–1201.7 ng/l	China	Li et al. (2015)
Groundwater and surface water	PAHs	—	Bangladesh	Mandal et al. (2015)
Surface water	Polychlorinated biphenyls (PCBs)	0.93–13.07 ng/l	China	Yang, Xie, Liu, and Wang (2015)
Wastewater and surface water	Alkylphenolic chemicals (APs)	—	USA	Barber et al. (2015)
Wastewater	Pharmaceutical residues	117 μg/l	South Africa	Matongo, Birungi, Moodley, and Ndungu (2015)
Surface water	Pharmaceutical residues	84.60 μg/l		
Surface water	Nonylphenol	694.6 ± 248.7 ng/l	China	Wang et al. (2015)
Groundwater	Nonylphenol	244.4 ± 230.8 ng/l		
Surface water	Nonylphenol (NP)	0.1–6.2 μg/l	Argentina	Babay, Itria, Ale, Becquart, and Gautier (2014)
	Mono‐ethoxylate (NP1EO)	0.1–9.2 μg/l		
	Di‐ethoxylate (NP2EO)	0.1–5.2 μg/l		
Wastewater	Pharmaceuticals and personal care products (PPCPs)	Influent: 7.26 μg/l Effluent: 6.72–940 ng/l	Spain	Carmona, Andreu, and Picó (2014)
Wastewater	Phthalate esters (PAEs)	6.95–61.49 ng/ml	China	Gao, Li, Wen, and Ren (2014)
Surface water	PAEs	9.93–45.55 ng/ml		
Drinking water sources	Di‐2‐ethylhexy phthalate (DEHP)	128.9–6570.9 ng/l	China	Liu, Chen, and Shen (2013)
	Di‐butyl phthalate (DBP)	52–4498.2 ng/l		
Freshwater	Dichlorodiphenyltrichloroethane (DDT) and its metabolites	Fall: 0.29 ± 0.69 ng/l Spring: 0.36 ± 0.91 ng/l	China	Wang et al. (2013)
Wastewater	Bisphenol A	0.07–1.68 μg/l	Canada	Mohapatra, Brar, Tyagi, and Surampalli (2011)
Surface water	PPCPs	56–1013 ng/l	South Korea	Yoon, Ryu, Oh, Choi, and Snyder (2010)
Surface water	Bisphenol A	Up to 330 ng/l	Netherlands	Belfroid, van Velzen, van der Horst, and Vethaark (2002)

The effect of EDC on the biochemical and physical integrity of water, as well as their impacts on the flora and fauna that depend on freshwaters, has been reported. These effects are profound in fish, wildlife, and humans. Some of the adverse effects in humans include infertility, increase in natal defects, alteration in sexual expression, and cancer (Jobling et al., 2013). Given the ecological risk and adverse health effects associated with exposure of humans to EDC, their removal from the environment should be the utmost priority of stakeholders.

Unfortunately, removal of EDC by most wastewater treatment plants seems to be inefficient as there is no specific unit designed to eliminate EDC in the present wastewater treatment technology (Zhang, Li, Wang, Niu, & Cai, 2016). Auriol, Filali‐Meknassi, Tyagi, Adams, and Surampalli (2006) made the following observations with the use of some conventional treatment processes for removal of EDC in wastewater: Coagulation with the use of “iron and aluminum salts” did not support any EDC removal; however, coagulation involving powdered activated carbon (PAC) removed a significant amount of “small‐sized contaminants” including EDC, while filtration processes, which allowed quite high EDC removal, are costly and involve a substantial maintenance in order to prevent membrane clogging. These and some other challenges have led to the development of different treatment methods for EDC removal employing the advanced oxidation processes such as photocatalysis, ozonation, the use of hypochlorites, and chlorine oxides (Silva, Otero, & Esteves, 2012; Taboada‐Puig et al., 2015). Although the advanced oxidation processes have recorded high EDC removal efficiency, they also present some challenges such as increased prices, narrow specificity, and generation of intermediates with unknown or higher estrogenic activity compared to their precursors (Oller, Malato, & Sanchez‐Perez, 2011; Silva et al., 2012; Taboada‐Puig et al., 2015). It is obvious that most of the conventional treatment methods are characterized by one challenge or the other. Consequently, research efforts should be channeled toward addressing these challenges and developing new wastewater treatment technologies that will effectively remove EDC and other emerging pollutants even at very low concentrations in wastewater. Hence, this paper discusses the potential of ligninolytic enzymes in the elimination of EDC in wastewater and proposes a new scheme of wastewater treatment process for EDC removal. Details of some reported methods of EDC removal are given in the succeeding section.

## 2 CONVENTIONAL METHODS FOR REMOVAL OF EDC IN WASTEWATER

Research efforts toward complete removal of EDC in wastewater have continued to increase, and these had led to appreciable progress in recent years. A major progress made so far include the development of new methods for removal of EDC in wastewater. Some of these methods include but not limited to adsorption, electrochemical oxidation, chemical advanced oxidation, photocatalysis, biodegradation, and enzymatic treatment. Details of these methods are presented as follow:

### 2.1 Adsorption by activated carbon

Adsorption by activated carbon is one of the most effective techniques for removal of EDC in wastewater (Jeirani, Niu, & Soltan, 2016; Nam, Choi, Kim, Her, & Zoh, 2014). This technique is based on hydrophobic interaction, which is determined by the nature of functional groups on the adsorbent and the adsorbates (Moreno‐Castilla, 2004). More so, formation of electron donor–acceptor complex and hydrogen bonding, as well as π–π dispersion interactions, are the major mechanisms reported for adsorption of organic pollutants by carbon in aqueous solutions (Li, Lei, & Huang, 2009; Lladó et al., 2015; Moreno‐Castilla, 2004). Jeirani et al. (2016), in a recent review, gave a concise documentation of the major mechanisms involved in adsorption of emerging pollutants on activated carbon.

A major progress in adsorption technique is the exploration of cheap adsorbents for adsorption of emerging organic pollutants. Ifelebuegu, Lester, Churchley, and Cartmell (2006) exploited coconut, wood, and coal‐based carbons for the removal of EE2 in wastewater final effluent with 99.3%, 96.4%, and 98.6% removal efficiency achieved, respectively. Krupadam, Sridevi, and Sakunthala (2011) employed crab shell chitin as a biosorbent for the removal of some EDC including benzo (a) anthracene, β‐estradiol, and BPA in contaminated groundwater. The use of chitin has, therefore, been suggested as a cost‐effective adsorbent for EDC elimination in aqueous solutions. Furthermore, Loffredo and Castellana (2015) conducted a comparative study on the efficiency of low‐cost adsorbents (almond shells and green compost) and ligninolytic fungi (*Pleurotus ostreatus* and *Sterenum hirsutum*) to remove organic pollutants (xenoestrogens and pesticides) from a landfill leachate. The study concluded that combined adsorption and biodegradation is suitable for the removal of xenoestrogens (BPA, ethinylestradiol, and 4‐n‐NP) and pesticides. Also, Saucier et al. (2015) assessed the use of microwave‐assisted carbon from cocoa shell as adsorbent for removal of sodium diclofenac and nimesulide (anti‐inflammatory drugs) in aqueous effluents. The study suggested that MWCS‐1.0 [a mixture of cocoa shell and inorganic components (CSC‐1.0) acidified with HCl] is capable of efficient removal of sodium diclofenac and nimesulide in simulated hospital effluents. Furthermore, Qin, Jia, Liu, Li, and Wu (2015) suggested metal‐organic frameworks with high porosity and large pore size as potential adsorbents for the removal of EDC in contaminated water.

### 2.2 Electrochemical oxidation

This approach combines electro‐enzymatic catalysis and electrocoagulation as a novel electrochemical approach for the removal of EDC in wastewater, with horseradish peroxidase (HRP) immobilized on graphite felt of titanium electrode as cathode and aluminum plate serving as anode of the working electrode (Zhao et al., 2015). Electrochemical approach has been used to remove BPA and reduce the total organic carbon (TOC) of wastewater. By this approach, Zhao et al. (2015) achieved 94% BPA removal and 52% TOC reduction in real wastewater upon sequencing treatment. The electro‐enzymatic process mediated polymerization of BPA and incorporation of BPA into humic acid (HA), thereby, transformed BPA. It also altered the chemical/structural features of HA, and this gave rise to a form more prone to electrocoagulation.

### 2.3 Chemical advanced oxidation

This approach involves the use of chemical oxidants for the removal of EDC in a process called advanced oxidative process (AOP), which is characterized by the generation of reactive oxygen species (ROS), primarily, hydroxyl radicals (˙OH) that, subsequently, oxidize organic pollutants to carbondioxide (CO_2_) and inorganic ions (Esplugas, Bila, Gustavo, Krause, & Dezotti, 2007) in wastewater. Another mechanism employed in this approach is the transformation of pollutants to some other metabolic products by some strong oxidizing agents such as chlorine (Cl_2_), chlorine dioxide (ClO_2_), hydrogen peroxide (H_2_O_2_), and ozone (O_3_) through oxidation–reduction reactions (Liu et al., 2009). The promising potentials of manganese oxide (MnO_2_) and calcium peroxide (CaO_2_) as oxidizing agents for EDC removal in wastewater have also been reported (Jiang, Huang, Chen, & Chen, 2009; Zhang, Wang, & Li, 2015). Han, Zhang, Zhao, and Feng (2015) synthesized a new class of stabilized MnO_2_ nanoparticles known as carboxymethyl cellulose‐stabilized MnO_2_ nanoparticles with potential for in situ “oxidative degradation of several emerging contaminants in soil and groundwater” (Han et al., 2015). More so, CaO_2_ oxidation has also been employed for effective removal of EDC including E1, E2, EE2, estriol, BPA, and 4‐NP in waste‐activated sludge (Zhang et al., 2015). However, the performance of CaO_2_ at removing the EDC was dose‐dependent (Zhang et al., 2015). In other words, the efficiency of EDC removal increased with CaO_2_ dosage. The ROS released during CaO_2_ oxidation have been identified as the major factor responsible for EDC removal, with hydroxyl radicals (˙OH) playing the most significant role. Interestingly, EDC products from CaO_2_ oxidation have shown less estrogenic activity than their precursors, which is an advantage over other advanced oxidation processes that may likely release by‐products with higher estrogenic activity than their precursors. CaO_2_ treatment has, therefore, been suggested as a promising technology for the removal of EDC in wastewater (Zhang et al., 2015).

Fenton oxidation is another chemical advanced oxidation employed for removal of EDC in wastewater. Fenton oxidation involves the use of ferrous salt and H_2_O_2_ to generate hydroxyl radicals with high redox potential for oxidation of a broad range of organic pollutants (Klavarioti, Mantzavinos, & Kassinos, 2009) including EDC. The effectiveness of this process is enhanced by ultraviolet irradiation in a photo‐Fenton reaction, which leads to generation of more hydroxyl radicals (Ifelebuegu & Ezenwa, 2011). Several studies have reported the use of Fenton oxidation in the degradation of organic compounds including pharmaceutical products (Mendez‐Arriaga, Esplugas, & Gimenez, 2010; Xu, Wang, Li, & Gu, 2004). Despite the effectiveness of Fenton oxidation, it is characterized by some major setbacks, which include narrow pH range of operation (pH 2–4) and recovery of dissolved ions from treated solutions, which require additional treatment step (Klavarioti et al., 2009). Furthermore, Fenton‐like oxidation has been employed for the removal of EDC in wastewater (Ifelebuegu & Ezenwa, 2011). Fenton‐like oxidation is the reaction of ferric ion generated from Fenton oxidation with H_2_O_2_ to generate ferrous ion and hydroxide radical, which is able to attack aromatic compounds that are protected against hydroxyl radical attack due to the natural organic matters that are present in the treatment plant (Lindsey & Tarr, 2000). Several studies have also reported the use of ozonation in the removal of EDC in water (Bila, Montalvao, & Dezotti, 2005; Huber, Canonica, Park, & von Gunten, 2003; Ternes et al., 2003; Vogna, Marotta, Napolitano, Andreozzi, & d'Ischia, 2004).

### 2.4 Photocatalysis

This mechanism removes EDC in a photochemical reaction catalyzed by semiconductor metal oxides known as photocatalysts such as titanium dioxide (TiO_2_), zinc oxide (ZnO), zinc sulfide (ZnS), ferric oxide (Fe_2_O_3_), and tin oxide (SnO_2_). During photocatalysis, photon energy absorbed by the catalyst produces an electron excitation, which leads to a change of level from valence to conduction band (Dalrymple, Yeh, & Trotz, 2007). Consequently, an electron–hole is created in the valence band. Therefore, the electron–hole moves to the surface of the catalyst, where it takes part in an oxidation–reduction reaction with the EDC or other emerging pollutants that are adsorbed on the catalyst. The hole may further interact with water (H_2_O) or H_2_O_2_ to produce hydroxyl radicals, which, subsequently, facilitate degradation of EDC or other micropollutants (Dalrymple et al., 2007; Wong & Chu, 2003) in an indirect photolysis. However, the efficiency of photocatalytic degradation is dependent on the absorbance spectrum of the pollutants, quantum yield of photocatalysis, concentration of H_2_O_2,_ and the water matrix (Klavarioti et al., 2009). Recent studies have reported the efficiency of photocatalytic degradation of EDC. Arlos et al. (2016) investigated the photocatalytic degradation of some target EDC including EE2, E2, E1, estriol, and BPA and their estrogenic activity by UV‐LED irradiated TiO_2._ All the compounds except E2 were efficiently degraded at a wide pH range as a significant reduction in the total estrogenic activity was also observed. Furthermore, previous studies have also reported reduction and removal of estrogenic activity by photocatalytic treatment with TiO_2_ (Coleman, Routledge, Sumpter, Eggins, & Byrne, 2004; Ohko et al., 2001, 2002).

### 2.5 Biodegradation

Biodegradation involves the use of microbes including fungi and bacteria for degradation of EDC and other environmental pollutants. Biodegradation has been described as a major removal mechanism that is capable of affecting the fate of EDC in the environment (Yu, Deeb, & Chu, 2013). Over the years, several studies have reported the degradation of various EDC by different microorganisms (Ahuactzin‐Perez et al., 2018; Cajthaml, 2015; Combalbert & Hernandez‐Raquet, 2010; Husain & Qayyum, 2012; Yu et al., 2013; Zhang et al., 2016; Zhao et al., 2016). Ligninolytic organisms, predominantly, white rot fungi (WRF), have received increased attention for degradation of various emerging micropollutants. There are quite a number of reviews on the potential of ligninolytic fungi in the efficient removal of EDC in the environment. Cabana, Jones, and Agathos (2007a) gave a comprehensive review on the capability of WRF to effectively eliminate EDC in various environmental matrices. In the review, the authors suggested the need to develop “robust and reliable biotechnological processes for the treatment of EDC‐contaminated environmental matrices” (Cabana, Jones, and Agathos, 2007a). Cajthaml (2015), in another review, reported the versatility of ligninolytic fungi in the degradation of EDC using the lignin‐modifying enzymes system and cytochrome P‐450. EDC degradation by ligninolytic fungi occurs through polymerization of the micropollutants or degradation of the original structure by extracellular enzymes system (Cajthaml, 2015). It is worthy of note that ligninolytic fungi are among the very few microbes with the ability to degrade EE2 and PCBs efficiently (Cajthaml, 2015).

Ligninolytic bacteria are also promising candidates for degradation of EDC, perhaps, because of their dexterity in the degradation of recalcitrant compounds and their abilities to produce some lignin‐modifying enzymes including laccase and manganese peroxidase, which are mostly responsible for the EDC degradation proficiency manifested by WRF. Bacteria seem to hold stronger potential for EDC degradation, given their striking resilience in diverse environments and the maneuverability of their genome. Furthermore, bacterial degradation has been suggested as an easy way to remove EDC in wastewater (Husain & Qayyum, 2012). To justify this claim, Zhang et al. (2016) gave a succinct documentation on the bacterial degradation of EDC and classified EDC‐degrading bacteria into the following: Proteobacteria, Firmicutes, Actinobacteria, and Bacteroidetes.

On the other hand, the combination of bacteria and physical methods such as adsorption is a novel technique with excellent potential for EDC elimination in an aqueous environment (Zhang et al., 2016). Nevertheless, a major concern on the adoption of biodegradation for EDC removal is the likely introduction of pathogenic microorganisms into the environment, which may also contribute to the problem of antibiotic and multiple drug resistance via horizontal gene transfer. Therefore, enzymatic treatment, involving the use of ligninolytic extracellular enzymes rather than the whole cell culture, will be a suitable alternative.

## 3 PROMISING LIGNINOLYTIC ENZYMES FOR EDC ELIMINATION

Laccases (EC 1.10.3.2), manganese peroxidase—MnP (EC 1.11.1.13), and versatile peroxidase—VP (EC 1.11.1.16) are ligninolytic enzymes (LEs) with promising potential for the removal of EDC in wastewater. The resourcefulness of LEs in the elimination of EDC is, perhaps, due to their high redox potentials for oxidation of a wide spectrum of organic compounds. Besides the lignin degradation activity of LEs, their potentials for bioremediation and wastewater effluent treatment have been reported (Husain, 2010; Mehta, 2012; Rajasundari & Murugesan, 2011). Furthermore, they have shown great potentials for the transformation of several types of recalcitrant aromatic compounds with known or suspected endocrine‐disrupting properties such as PAHs, PCBs, APs, and pesticides (Davila‐Vazquez, Tinoco, Pickard, & Vazquez‐Duhalt, 2005; Garcia‐Morales et al., 2015; Mao, Lu, Gao, & Huang, 2010; Suzuki, Hirai, Murata, & Nishida, 2003; Taboada‐Puig et al., 2011; Touahar, Haroune, Ba, Bellenger, & Cabana, 2014).

The potential of laccase and MnP for removal of EDC in wastewater has been well studied (Auriol et al., 2008; Ba, Jones, & Cabana, 2014; Cabana, Jones, & Agathos, 2007b, 2009; Kim, Yeo, Kim, & Choi, 2008; Lloret et al., 2012; Sei, Takeda, Soda, Fujita, & Ike, 2007). However, there is dearth of information on the use of VP for EDC elimination. Among the LEs, VP seems to be the most promising for the elimination of EDC in wastewater, given its peculiar attribute of hybrid molecular architecture. Besides, unlike laccase, VP is not dependent on redox mediators for degradation of micropollutants (Ravichandran & Sridhar, 2016). Summary of EDC removal by LEs is presented in Table 2.

**Table 2:** EDC removal by ligninolytic enzymes

Reaction matrix	Classes of EDC	Removal efficiency (%)	Enzyme used	References
Aqueous system	Bisphenol A	≈100	Immobilized laccase	Zdarta et al. (2018)
	Bisphenol F	≈100		
	Bisphenol S	>40		
Aqueous system	Acetaminophen	90	Immobilized laccase	Garcia‐Morales et al. (2018)
	Diclofenac	68		
Aqueous system	Bisphenol A	90	Immobilized laccase	Ji et al. (2017)
	Carbamazepine	40		
Aqueous system	Bisphenol A	100	Crude laccase	de Freitas et al. (2017)
Aqueous system	Bisphenol A	100	Laccase with mediator (Hydroxybenzotriazole)	Daasi et al. (2016)
Wastewater	Bisphenol A (BPA)	100	Versatile peroxidase using two‐stage system (TSS)	Taboada‐Puig et al. (2015).
	Triclosan			
	Estrone (E1)			
	17β‐estradiol (E2)			
	17α‐ethinylestradiol (EE2)			
Synthetic and groundwater	Bisphenol A	89	Free laccase cocktail	Garcia‐Morales et al. (2015)
	4‐nonylphenol	93		
	17α‐ethinylestradiol	100		
	Triclosan	90		
Wastewater	Nonylphenol	99.2	Versatile peroxidase using TSS	Mendez‐Hernandez et al. (2015)
Aqueous system	Nonylphenol and triclosan	>95%	Laccase	Ramírez‐Cavazos et al. (2014)
Wastewater	Acetaminophen	93	Cross‐linked laccase aggregates and polysulfone hollow fiber microfilter membrane	Ba et al. (2014)
	Mefenamic acid			
	Carbamazepine			
Aqueous system	Bisphenol A	100	Immobilized laccase	Debaste et al. (2014)
	Nonylphenol			
	Triclosan			
Wastewater	Estrone	83.6	Laccase using enzymatic membrane reactor (EMR)	Lloret, Eibes, Moreira, Feijoo, and Lema (2013)
	17β‐estradiol (E2)	94		
	17α‐ethinylestradiol (EE2)	93.6		
Water	Bisphenol A	90	Encapsulated ligninolytic enzymes (Manganese peroxidase, lignin peroxidase, and laccase)	Gassara et al. (2013)
Aqueous system	Bisphenol A	80	Immobilized laccase	Songulashvili et al. (2012)
	Nonylphenol	40		
	Triclosan	60		
Aqueous system	Estrone	65	Immobilized laccase in a packed‐bed reactor	Lloret et al. (2012)
	17β‐estradiol (E2)	80		
	17α‐ethinylestradiol (EE2)	80		
Aqueous system	Diclofenac and estrogen hormones	100	Versatile peroxidase	Eibes, Debernardi, Feijoo, Moreira, and Lema (2011)
	Sulfamethoxazole and Naproxen	80		
Wastewater	Bisphenol A, B, F	100	Immobilized laccase	Diano and Mita (2011)
Aqueous system	Triclosan	99.4	Manganese peroxidase	Inoue et al. (2010)
Wastewater	Estrone	100	Laccase	Auriol et al. (2008)
	Estriol			
	17β‐estradiol (E2)			
	17α‐ethinylestradiol			
Simulated wastewater	Nonylphenol	100	Laccase	Cabana, Jiwan, et al. (2007)
	Bisphenol A	100		
	Triclosan	65		
Simulated wastewater	Nonylphenol	100	Immobilized laccase in fluidized bed reactor	Cabana, Jones, and Agathos (2007b)
	Bisphenol A			
	Triclosan			
Aqueous system	Natural steroidal hormone, estrone	98	Manganese peroxidase and laccase	Tamagawa et al. (2006)
Aqueous system	Genistein	93	Manganese peroxidase and laccase	Tamagawa, Hirai, Kawai, and Nishida (2005)
Aqueous system	Bisphenol A	100	Manganese peroxidase and laccase‐1‐hydroxybenzotriazole (laccase‐HBT) system	Tsutsumi et al. (2001)
	Nonylphenol			

### 3.1 Laccases (EC.1.10.3.2)

Laccases are multicopper oxidases (MCOs) that oxidize a wide range of aromatic compounds using molecular oxygen in a radical‐catalyzed reaction (Strong & Claus, 2011). Usually, their molecular weights (MW), optimal pH, and temperature fall within 58–90 kDa, 2–10, and 40–65°C, respectively (Quaratino, Federici, Petruccioli, Fenice, & D'Aannibale, 2007; Zouari‐Mechichi et al., 2006).

They are the largest member of MCOs family, with wide distribution in eukaryotes and prokaryotes (Sirim, Wagner, Wang, Schmid, & Pleiss, 2011). However, microbial laccases have attracted much interest, probably, owing to their low substrate specificity and capability to oxidize different compounds (Gasser, Ammann, Shahagaldian, & Corvini, 2014). Other MCOs include ferroxidases, ascorbate oxidase, and ceruloplasmin (Strong & Claus, 2011). MCOs oxidize their substrates with a concomitant four‐electron reduction of molecular oxygen to water (Sirim et al., 2011). They are divided into three classes, based on their copper centers: type 1 (blue), type 2 (normal), and type 3 or coupled binuclear (Messerschmidt & Huber, 1990; Ouzounis & Sander, 1991). Type 1 (T1) and type 2 (T2) have one Cu atom each, while type 3 (T3) has two Cu atoms (Wong, 2009).

The catalytic efficiency of laccases is dependent on the redox potential of the active site type 1 (T1) copper ion (Eldridge et al., 2017), where substrate oxidation occurs in a one‐electron reaction. Usually, microbial laccases exhibit higher redox potential than laccases of plant origin (Gasser et al., 2014). This indicates that laccases from microbes may probably have higher activity and catalytic efficiency when compared to plant laccases. In the other, T2 and T3 form a trinuclear cluster, T2/T3, where molecular oxygen is reduced to water through the electrons transferred from T1 site to the trinuclear site (Gasser et al., 2014; Wong, 2009). Generally, the catalytic reaction of laccases involves oxidation of four molecules of substrate and reduction of molecular oxygen to two water molecules (Gasser et al., 2014). The use of atmospheric oxygen as electron acceptor in a laccase‐catalyzed reaction is an advantage over the use of hydrogen peroxide by peroxidases. Nonetheless, laccases depend on redox mediators such as 2,2′‐azino‐bis (3‐ethylbenzthiazoline‐6‐sulfonic acid) for degradation of nonphenolic compounds. Despite this shortcoming, laccases have attracted considerable attention in the last one decade, probably, owing to their potential applications in various biotechnological processes such as biopulping, biobleaching, bioremediation, juice/wine clarification, textile dye decolorization, degradation of xenobiotics, and effluent treatment (Afreen, Anwer, Singh, & Fatma, 2016; Chandra & Chowdhary, 2015; Couto & Toca Herrera, 2006). Interestingly, there has been a drastic shift from the conventional application of laccases in lignin modification to degradation of emerging micropollutants.

### 3.2 Manganese peroxidase (EC.1.11.1.13)

MnP was discovered by Kuwahara, Glenn, Morgan, and Gold (1984) and is the most common ligninolytic peroxidase produced by microbes (Hofrichter, 2002). Its participation in lignin modification has been documented and studied extensively in fungi (Hofrichter, 2002). Nevertheless, there is rarity of information on MnP‐producing bacteria. MnP catalytic reaction involves oxidation of Mn^2+^ to Mn^3+^, which then oxidizes a broad spectrum of phenolic substrates including phenolic lignin monomers (Tuor, Wariishi, Schoemaker, & Gold, 1992). The Mn^3+^ formed from the oxidation of Mn^2+^ present in lignocellulosic materials is stabilized by reacting with a carboxylic acid such as tartrate which serves as ion chelator. The resultant complex will in turn oxidize the phenolic component of lignin structure which leads to generation of unstable radicals that may breakdown naturally (Hofrichter, 2002). However, MnP is also capable of oxidizing nonphenolic compounds, but with the involvement of redox mediators such as thiyl or lipid radicals (Abdel‐Hamid, Solbiati, & Cann, 2013; Reddy, Sridhar, & Gold, 2003). More so, the ability of MnP to oxidize and degrade lignin and other recalcitrant compounds has been reported (Bogan, Lamar, & Hammel, 1996; Dehorter & Blondeau, 1993; Hofrichter, 2002; Hofrichter, Steffen, & Hatakka, 2001; Hofrichter, Ullrich, Pecyna, Liers, & Lundell, 2010). Generally, MnP has a MW ranging from 38 to 62.5 kDa, with most purified MnPs having MW of about 45 kDa (Hatakka, 1994). About 11 various isoforms of MnP have been identified in *Ceriporiopsis subvermispora* (Lobos, Larram, Salas, Cullen, & Vicuna, 1994) with variations in the isoelectric point of the different isoforms.

### 3.3 Versatile peroxidase (EC.1.11.1.6)

VP belongs to the group of enzymes called ligninases, which are microbial extracellular enzymes capable of degrading lignin. Generally, ligninases are characterized by high redox potential for oxidation of phenolic and nonphenolic compounds. As well, they have shown capability for degradation of some recalcitrant compounds including “chlorophenols, polycyclic aromatic hydrocarbons (PAHs), organophosphorus compounds, and phenols” (Wesenberg, Kyriakides, & Agathos, 2003), thus informing their potentials for diverse industrial and biotechnological applications (Falade et al., 2017; Husain, 2010; Mehta, 2012; Rajasundari & Murugesan, 2011).

VP is a relatively new and unique lignin‐modifying heme peroxidase belonging to the same class (class II of peroxidase–catalase superfamily) with lignin peroxidase (LiP) and MnP. It is also referred to as “hybrid peroxidase” or “lignin‐manganese peroxidase” and is largely produced by ligninolytic fungi belonging to certain genera: *Bjerkandera* (Heinfling, Martınez, Martınez, Bergbauer, & Szewzyk, 1998), *Pleurotus* (Palma, Lloret, Sepulveda, & Contreras, 2016; Ruiz‐Duenas, Martınez, & Martınez, 1999), and *Lepista* (Zorn, Langhoff, Scheibner, Nimtz, & Berger, 2003). Its production by *Phanerochaete chrysosporium* has also been reported (Coconi‐Linares et al., 2014). Nonetheless, there is limited information on its production by bacteria. Thus, exploitation of bacteria and other fungal genera for VP production is imperative.

**Figure 1:**
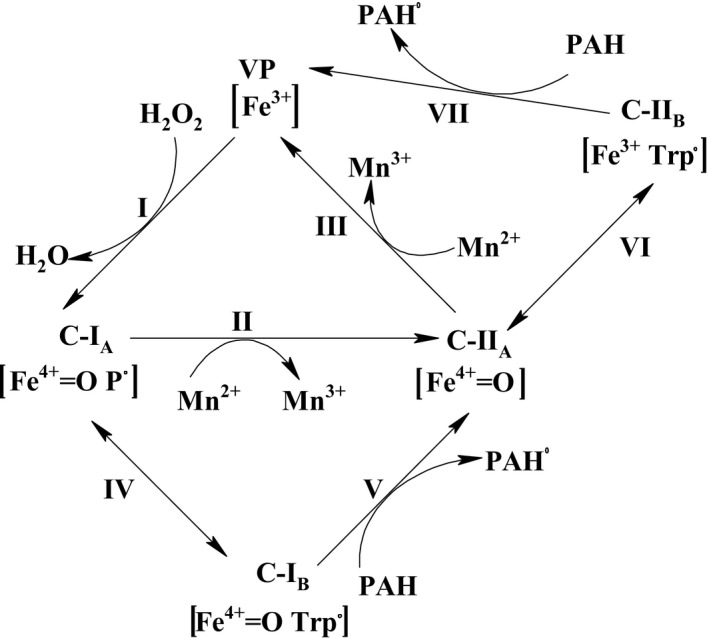
Exploitation of the VP catalytic cycle for EDC removal. The VP catalytic cycle is adapted from Perez‐Boada et al. (2005) with permission from Elsevier (license number: 4365891195222). C‐I_A_ (Compound I_A_, containing Fe^4+^‐oxo and porphyrin cation radical), C‐II_A_ (Compound II_A_, containing Fe^4+^‐oxo after reduction in porphyrin), C‐I_B_ (Compound I_B_, containing Fe^4+^‐oxo and tryptophanyl (Trp^164^) radical), C‐II_B_ (Compound II_B_, containing Fe^3+^ and tryptophanyl (Trp^164^) radical), and PAH (polycyclic aromatic hydrocarbons). I–III: Reactions involved in Mn^2+^ oxidation mechanism for EDC removal. Mn^3+^ generated in step III forms a complex with a dicarboxylic acid such as oxalate/malonate/tartrate, which is responsible for subsequent degradation of EDC. I, IV‐VII: Reactions involved in long‐range electron transfer mechanism proposed for EDC removal. The tryptophanyl radical generated on the surface of C‐II_B_ could be exploited for degradation of EDC such as PAH in step VII

The uniqueness of VP is manifested in its hybrid molecular architecture, which combines different substrate binding and oxidation sites (Camarero, Sarkar, Ruiz‐Duenas, Martinez, & Martinez, 1999). Its characteristic oxidation of high and low redox potential substrates motivates for its potential application in the elimination of EDC in wastewater. Furthermore, the enzyme has the ability to combine the substrate specificity of two ligninolytic peroxidases (MnP and LiP) and one other fungal peroxidase family, *Coprinopsis cinerea* peroxidase (CIP) (Perez‐Boada, Ruiz‐Duenas, Pogni, Basosi, & Choinowski, 2005). Consequently, it is capable of oxidizing a wide range of substrates such as Mn^2+^, phenolic and nonphenolic lignin model dimers, α‐keto‐γ‐thiomethylbutyric acid (KTBA), veratryl alcohol (VA), dimethoxybenzenes, synthetic dyes, substituted phenols, and hydroquinones (Caramelo, Martınez, & Martınez, 1999; Perez‐Boada et al., 2005). It employs the MnP pathway by oxidizing Mn^2+^ to Mn^3+^ with H_2_O_2_ as electron acceptor (Figure 1); however, Mn^3+^ is highly reactive, but has a very short half‐life. Thus, when VP is utilizing the MnP pathway, a dicarboxylic organic acid such as oxalate, tartrate, or malonate is required to form a stable complex with Mn^3+^ (Mn^3+^‐oxalate, Mn^3+^‐tartrate, or Mn^3+^‐malonate). With the utilization of this mechanism, VP is capable of oxidizing pollutants situated far away from it by the action of the metallic ion complex (Taboada‐Puig et al., 2015). The utilization of MnP pathway by VP commits it to oxidation of phenolic substrates as it is also able to oxidize nonphenolic compounds and other typical substrates of LiP using the normal LiP catalytic reaction mechanism. However, LiP is able to oxidize veratryl alcohol, a typical LiP substrate more effectively than VP. The variation in the catalysis of LiP and VP has been attributed to the variation in the tryptophan environment of the enzymes (Khindaria, Yamazaki, & Aust, 1996).

VP also employs the “long‐range electron transfer (LRET) mechanism in the oxidation of high redox potential aromatic compounds” (Perez‐Boada et al., 2005; Ruiz‐Dueñas et al., 2009). Specifically, three possible LRET pathways for the oxidation of high redox potential aromatic compounds have been revealed in two VP isozymes (VPL and VPS 1) of *Pleurotus eryngii* (Caramelo et al., 1999; Perez‐Boada et al., 2005; Ruiz‐Duenas et al., 1999). The pathways start at either TrP 164 or His 232 of VPL and at His 82 or TrP 170 of VPS 1, which is homologous to TrP 164 in VPL (Perez‐Boada et al., 2005; Ruiz‐Dueñas et al., 2009). Furthermore, the involvement of TrP 164 in the oxidation of veratryl alcohol and reactive black 5 has been reported. However, the other two pathways (His 232 and His 82) were not involved in LRET (Perez‐Boada et al., 2005). Therefore, the ability of VP to oxidize high redox potential compounds could, perhaps, be linked to an exposed catalytic tryptophan: Trp‐164, which forms a radical on the surface of the enzyme through a LRET to the heme (Ruiz‐Dueñas et al., 2009; Saez‐Jimenez et al., 2015). Hence, LRET could suffice as a novel mechanism for EDC removal by VP.

## 4 POTENTIAL OF LEs IN THE ELIMINATION OF EDC IN WASTEWATER

The high environmental and health risk posed by exposure of human to EDC and the inefficiency of the conventional treatment approaches for complete removal of EDC in wastewater, as well as some challenges that characterized the conventional treatment processes, have led to an increased interest in the exploration of alternative treatment processes for elimination of EDC in wastewater. Therefore, enzymatic treatment process, involving the use of ligninolytic oxidative enzymes for EDC removal, has recently attracted attention as an environmentally friendly alternative. The potential of some LEs including laccase, MnP, and VP for efficient removal of EDC in water has recently been reported (Diano & Mita, 2011; Garcia‐Morales et al., 2015; Ramírez‐Cavazos et al., 2014; Taboada‐Puig et al., 2011; Touahar et al., 2014; Wen, Jia, & Li, 2009, 2010; Zhang & Geissen, 2010).

Immobilized laccase from *Cerrena unicolor* C‐139 eliminated 80% of BPA, 40% of NP, and 60% of triclosan from solutions that contained 50 μmol of each endocrine disruptor, respectively (Songulashvili, Jimenez‐tobon, Jaspers, & Penninckx, 2012). Also, Debaste, Songulashvili, and Penninckx (2014) reported total removal of BPA, 4‐NP, and triclosan by immobilized laccase from *Cerrena unicolor*. The authors, therefore, suggested the potential application of the immobilized enzyme for elimination of harmful micropollutants industrially and domestically. On the other hand, Eldridge et al. (2017) have attributed the efficient removal of EE2 by *Lentinula edodes* (Shiitake) to laccase as the induction of laccase production in the organism increased the removal efficiency of the pollutant from 50% to 80%. More so, Zdarta et al. (2018) reported approximately 100% removal of BPA and bisphenol F (BPF) by *Trametes versicolor* laccase immobilized on *Hippospongia communis* spongin scaffolds. However, the removal efficiency of the enzyme on bisphenol S (BPS) was only greater than 40%. The removal efficiency of the enzyme was optimal at slightly acidic pH (4–5), while the optimum temperature ranged from 30 to 40°C.

A new approach for elimination of EDC in wastewater is the immobilization of LEs on nanoparticles. Garcia‐Morales et al. (2018) reported 90% and 68% biotransformation of acetaminophen and diclofenac by *Pycnoporus sanguineus* laccase immobilized onto titania nanoparticle, respectively. Likewise, *Pleurotus ostreatus* crude laccase immobilized on functionalized TiO_2_ nanoparticles attained 90% BPA degradation within 6 hr of treatment, while only about 10% degradation efficiency was recorded with carbamazepine after 48 hr (Ji, Nguyen, Hou, Hai, & Chen, 2017). The poor elimination of carbamazepine in the study was attributed to the high redox potential of the compound, which hampered its oxidation by the enzyme (Hata, Shintate, Kawai, Okamura, & Nishida, 2010; Ji et al., 2017). Nevertheless, degradation of carbamazepine was enhanced in the presence of BPA, with 40% elimination attained after 24 hr reaction. The authors, therefore, concluded that oxidative products of BPA had a redox mediator effect on the degradation of carbamazepine. The findings from the study suggested that the “presence of more reactive micropollutant can promote the removal of the more recalcitrant pollutants” in a cocktail (Ji et al., 2017). Also, crude laccases from *P. ostreatus* and *P. pulmonarius* were able to degrade BPA in aqueous solution, with 100% and 85% degradation efficiency achieved, respectively, within 1 hr (de Freitas et al., 2017). Moreover, *P. ostreatus* laccase reduced BPA toxicity from 85% to less than 5%, but there was no decrease in toxicity when treated with laccase from *P. pulmonarius*. The study indicated that degradation of BPA by *P. pulmonarius* laccase, probably, generated metabolites with the same toxicity as the parent compound (de Freitas et al., 2017). Therefore, crude laccase from *P. ostreatus* was recommended as an efficient degrader of EDC. Besides the use of laccase alone for EDC elimination, Gassara, Brar, Verma, and Tyagi (2013) assessed the effectiveness of free LEs (Laccase, MnP and LiP) and encapsulated LEs in the degradation of BPA. The authors recorded higher degradation efficiency (90%) when the three LEs were encapsulated on polyacrylamide hydrogel and pectin, while only 26% efficiency was observed with the free enzymes.

MnP is another ligninolytic enzyme that has shown effectiveness for elimination of EDC (Inoue, Hata, Kawai, Okamura, & Nishida, 2010; Tamagawa, Yamaki, Hirai, Kawai, & Nishida, 2006; Tsutsumi, Haneda, & Nishida, 2001). In a study by Tsutsumi et al. (2001), BPA and NP were treated with MnP and laccase from ligninolytic fungi. MnP was able to completely remove the target compounds in an aqueous solution after 1 hr of treatment, but not without estrogenic activities. Upon extension of treatment time to 12 hr, the observed estrogenic activities were totally removed. Similarly, Inoue et al. (2010) treated triclosan with MnP from *P. chrysosporium*,* T. versicolor* laccase, and the laccase (0.5 and 2.0 nkat/ml) with 1‐hydroxybenzotriazole (0.2 mM) as mediator. The authors observed 99.4% triclosan removal by MnP after 1 hr of treatment, while 10.2% and 29% elimination were observed with the use of laccase and laccase–mediator system, respectively. The ability of MnP to almost remove the target compound completely within a short period indicates its potential for degradation of other classes of EDC.

VP has recently emerged with promising potential for elimination of EDC in wastewater. One of the few reports that implicated VP in EDC removal is the work of Taboada‐Puig et al. (2011). They produced a combined cross‐linked enzyme aggregate from VP and glucose oxidase (combined CLEA) and investigated its ability to eliminate the following endocrine disruptors: BPA, NP, triclosan, EE2, and E2. Coaggregation of VP with glucose oxidase resulted in an increased activity recovery of 89% from the initial activity of 67% and an increased stability of VP against H_2_O_2_. The combined CLEA was able to remove all the target pollutants except triclosan, while the removal of their estrogenic activities was more than 55% for all the EDC except triclosan. The exploration of other H_2_O_2_‐producing enzymes with more appropriate substrates in water treatment rather than glucose in the case of glucose oxidase has been suggested as glucose may support the unwanted growth of microorganisms. Adoption of this concept (coaggregation of glucose oxidase with other ligninolytic peroxidases) is desirable for EDC removal and other applications as this will nullify the cost of H_2_O_2._ Likewise, Touahar et al. (2014) investigated the ability of a combined cross‐linked enzyme aggregate (combi‐CLEA) {comprising laccase, VP, and glucose oxidase} to transform a cocktail of pharmaceutically active compounds (PhACs) in a mixed solution and synthetic wastewater. The free enzymes and combi‐CLEA showed the ability to efficiently transform nonsteroidal anti‐inflammatory drugs (acetaminophen, naproxen, mefenamic acid, diclofenac, and indometacin) in a mixed solution and eliminate acetaminophen in municipal wastewater. However, combi‐CLEA exhibited more improved removal efficiency. The study also demonstrated that VP had a wider removal spectrum than laccase. Furthermore, Taboada‐Puig et al. (2015) utilized the oxidant, Mn^3+^‐malonate generated by VP in a two‐stage (TS) system for continuous removal of the following EDC: BPA, triclosan, E1, E2, and EE2 from synthetic and real wastewaters at degradation rates ranging from 28 to 58 μg/L min, with little enzyme inactivation observed. Interestingly, a 14‐fold increase in the EDC removal efficiency of VP in a TS system was observed when compared with a regular enzymatic membrane reactor (EMR) system. Also, some of the operational challenges encountered during EDC removal in an EMR system were prevented, as the TS system was able to separate the complex formation stage from the contaminant oxidation stage. It is noteworthy that VP in a TS enzymatic system exhibited 100% removal efficiency for all the EDC studied, therefore demonstrating the practicability of this approach for removing EDC at both high and environmental concentrations.

## 5 PROPOSED SCHEME OF WASTEWATER TREATMENT PROCESS FOR EDC REMOVAL BY LEs

**Figure 2:**
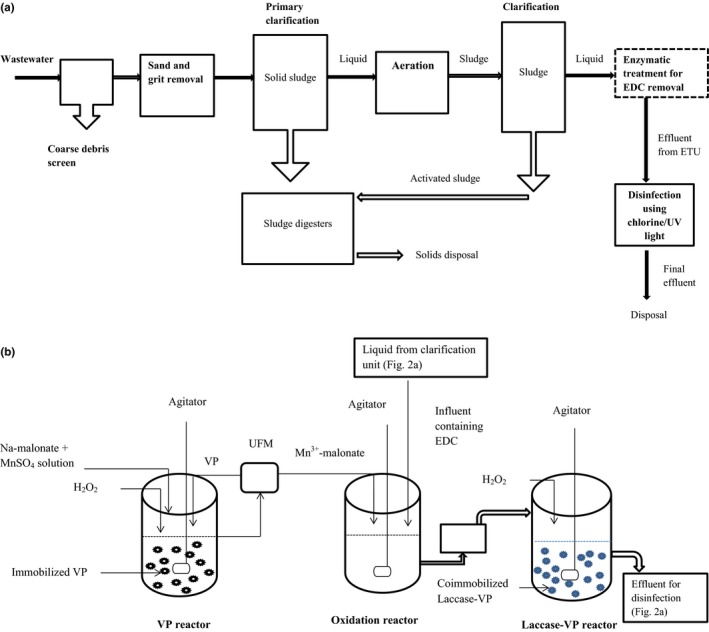
(a) Proposed scheme of wastewater treatment process for EDC removal by LEs. Adapted from https://www.britannica.com/technology/wastewater-treatment. Primary Treatment Stage: coarse debris screen, sand and grit removal, and primary clarification. Secondary Treatment Stage: aeration and clarification. Tertiary Treatment Stage: enzymatic treatment for EDC removal and disinfection. Solid lines: conventional treatment units; broken lines: proposed additional treatment unit. ETU: enzymatic treatment unit. (b) Proposed treatment stages for EDC elimination by LEs using continuous stirred tank reactors. Stage I (VP reactor): generation of Mn^3+^‐malonate complex by immobilized VP via the MnP mechanism. Stage II (oxidation reactor): oxidation of EDC by Mn^3+^‐malonate complex. Stage III (laccase‐VP reactor): treatment of residual EDC with coimmobilized laccase‐VP. Stages I and II are adapted from Mendez‐Hernandez et al. (2015). UFM: ultrafiltration membrane

The present wastewater treatment technology involves three different stages including primary, secondary, and tertiary treatments. Each stage has specific units for specified treatment (Figure 2a). In most cases, the tertiary wastewater treatment stage involves disinfection and probably, nutrient removal (optional). However, no specific unit is designed to remove EDC during wastewater treatment process. This deficiency has, perhaps, resulted in the occurrence of EDC in wastewater treatment plant effluents (Huang et al., 2014; Ifelebuegu, 2011; Martın, Camacho‐Munoz, Santos, Aparicio, & Alonso, 2012; Pessoa et al., 2014; Ra et al., 2011; Zhang et al., 2016).

This paper, therefore, proposes a scheme of wastewater treatment process that includes a specific unit for EDC removal at the tertiary treatment stage (Figure 2a). Upon clarification, the liquid is passed through enzymatic treatment unit (ETU) for EDC removal: a three‐stage continuous stirred tank reactor (Figure 2b). The first stage is the VP reactor, where Mn^3+^‐dicarboxylic acid complex (Mn^3+^‐malonate, Mn^3+^‐tartrate, or Mn^3+^‐oxalate) is generated by immobilized VP, while the second stage (oxidation reactor) involves the oxidation of EDC by the complex generated in stage I (Mendez‐Hernandez et al., 2015). In the third stage (laccase‐VP reactor), effluent from the oxidation reactor is further treated with laccase coimmobilized with VP to eliminate any residual EDC before passing the effluent through disinfection unit and subsequent disposal. During the treatment process, the VP reactor will be fed with two different peristaltic pumps. One will be used for the enzyme activator, H_2_O_2_, while the other will be used for the solution of sodium malonate and MnSO_4_ (Mendez‐Hernandez et al., 2015) at predetermined feeding rates. Another peristaltic pump will be used to feed the laccase‐VP reactor with H_2_O_2._ However, further research is required to optimize the concentrations of H_2_O_2_, Mn^2+^, and sodium malonate required by VP. It is also imperative to regulate the concentration of H_2_O_2_ in the laccase‐VP reactor to ensure that laccase activity is not adversely affected as increase in H_2_O_2_ may inhibit the enzyme activity (Milton, Giroud, Thumser, Minteer, & Slade, 2013). However, addition of H_2_O_2_ may also increase laccase activity during oxidation of some phenolic compounds (Min, Kim, Kim, Jung, & Hah, 2001). Although both enzymes usually perform optimally in slightly acidic pH region (Jarosz‐Wilkolazka, Luterek, & Olszewska, 2008; Min et al., 2001; Zdarta et al., 2018), it is important to determine the pH requirements of the enzymes to ensure best performance during application. Likewise, efforts should be geared toward assessing the potential toxicity of Mn^2+^ and malonate in the effluent and the chance of recovering them after the enzymatic treatment.

The use of immobilized enzymes is suggested for the proposed technology as immobilization increases enzyme stability and allows the enzymes to be reused in subsequent treatment (Zdarta et al., 2018). A three‐stage reaction system is necessary to ensure high efficiency in the elimination of EDC during treatment process. One of the benefits of separating the oxidation process from the Mn^3+^ complex generation system is that it allows recirculation of the immobilized enzyme into the VP reactor for reuse (Taboada‐Puig et al., 2015). Also, it prevents some operative challenges that characterize conventional enzymatic reactors such as decrease in enzyme activity against time (Rios, Belleville, Paolucci, & Sanchez, 2004; Taboada‐Puig et al., 2015). Moreover, a second enzymatic reactor with coimmobilized laccase and VP is essential for complete EDC removal as there is possibility that some more recalcitrant EDC, which may resist oxidation by Mn^3+^ complex in stage II (Figure 2b), are present. However, coimmobilized laccase‐VP will have a wider EDC removal spectrum through combined advantages. At this stage, VP will catalyze EDC removal in Mn‐independent reaction through LRET mechanism, a typical LiP catalytic pathway. Exploitation of the LiP catalytic mechanism by VP will ensure degradation of nonphenolic micropollutants.

## 6 CONCLUSION

Indeed, LEs have shown great potential for degradation of EDC and other emerging organic micropollutants in wastewater, hence their potential applications in bioremediation and the water sector. More so, a new design of wastewater treatment technology that includes a three‐stage continuous stirred tank reactor for EDC removal should be adopted as this will prevent discharge of the micropollutants directly into freshwater environment. Furthermore, coimmobilization or combined cross‐linking of laccase and VP will be a promising approach for complete elimination of EDC and other emerging organic pollutants in wastewater as this will provide leverage for laccase in the degradation of nonphenolic pollutants and, consequently, nullifying the cost of redox mediators required by laccase for degradation of nonphenolic compounds.

## CONFLICT OF INTEREST

Authors declare that there is no conflict of interests.

## AUTHORS’ CONTRIBUTIONS

Ayodeji O. Falade conceptualized the manuscript and wrote the first draft. Leonard V. Mabinya, Anthony I. Okoh, and Uchechukwu U. Nwodo contributed to the conception of the manuscript and thoroughly reviewed the manuscript. All authors approved the final version of the manuscript.

## DATA ACCESSIBILITY

No new data were generated in support of this review article.
